# COVID-19: The Current Situation in the Democratic Republic of Congo

**DOI:** 10.4269/ajtmh.20-1169

**Published:** 2020-10-09

**Authors:** Carl Agisha Juma, Nestor Kalume Mushabaa, Feruzi Abdu Salam, Attaullah Ahmadi, Don Eliseo Lucero-Prisno

**Affiliations:** 1 Global Health Focus Africa, Bukavu, The Democratic Republic of Congo;; 2 Department of Gaenecology, University of Goma, Goma, The Democratic Republic of Congo;; 3 Health Maintenance Organization in Africa, Goma, The Democratic Republic of Congo;; 4 Kabul University of Medical Sciences, Kabul, Afghanistan;; 5 Department of Global Health and Development, London School of Hygiene and Tropical Medicine, London, United Kingdom;; 6 Faculty of Management and Development Studies, University of the Philippines (Open University), Los Baños, Philippines

## Abstract

COVID-19 is a highly contagious disease that has affected all African countries including the Democratic Republic of Congo (DRC). Formidable challenges limit precautionary measures which were instituted by the government to curb the pandemic. Insufficient COVID-19 testing laboratories, limited medical and personal protective equipment, and an inadequate number of health workers leave the country ill-equipped in the fight against the pandemic. Lack of assistance from the government to those who lost their jobs due to lockdown forced these individuals to go outside to find provisions, thus increasing the spread of the virus. Moreover, the fragile healthcare system is overburdened by civil conflicts and other epidemics and endemics amid the COVID-19 pandemic. The conflicts have led to thousands of deaths and hundreds of thousands of displacements and deprived many people of basic health services. The 11th outbreak of Ebola has been increasing at an alarming pace, and it is expected to soar because of a shortfall of funds and insufficient numbers of health workers. The DRC with the cooperation of regional powers needs to address these challenges in a manner similar to that used in the previous Ebola epidemics. Moreover, the government should have a balance in shifting the available resources between COVID-19 and other diseases. Until a vaccine is available, the DRC needs to be prudent when lifting restrictions to prevent explosion of new cases.

## INTRODUCTION

COVID-19 is a global public health emergency of international concern^1^ which has spread to many countries around the world including African countries.^2^ The first case of COVID-19 in the Democratic Republic of Congo (DRC) was a Congolese returnee from France, identified on March 10, 2020 in Kinshasa.^3^ Since then, the number of confirmed cases increased. There have been 10,630 confirmed cases and, 272 deaths recorded as of September 28, 2020.^4^ Kinshasa is more highly affected than any other province with 8,138 confirmed cases, followed by North-Kivu, Kongo Central, and Haut Katanga.

After the first few cases, the Congolese government declared a state of emergency and set up a multi-sectoral national committee to devise strategies to address the pandemic. As part of the strategies, lockdown was first enforced in Kinshasa and then across the country. Raising people’s awareness and imposing restrictions on the borders were initiated.^3^ Flights from COVID-19–infected countries were suspended,^5^ and Congolese returnees were recommended to stay on a 14-day self-quarantine. Schools and universities were shut, and mass gatherings of more than 20 individuals were prohibited.^5^ Wearing masks became compulsory outside houses.

## COMMENTARY

The DRC was expected to deal effectively with the pandemic by taking advantage of preventive lessons learned from its previous epidemics. However, the current indicators are dire. This article aims to provide a critical commentary on the formidable challenges which have hampered the COVID-19 control efforts and overwhelmed the already weak healthcare system.

The number of new cases in the DRC is continuously increasing, with a test positivity rate of 21%.^6^ The country remains ill-equipped to address COVID-19. There is shortage of facilities to deal with the pandemic. Of 26 provinces, COVID-19 testing is only available in the towns of Kinshasa, Matadi, Lubumbashi, Goma, Kolwezi, and Mbandaka.^7^ The daily testing of only 900 is very low compared with a population of more than 100 million.^8^ The National Institute of Biomedical Research (NIBR) is the main testing laboratory of the country based in Kinshasa. Transportation of samples from remote places to the NIBR has been delayed because of inadequate logistical resources and unpaved roads.^3^ There are also insufficient medical and personal protective equipment and diagnostic devices.^3^ Moreover, poor sanitation and a dearth of clean water leave Congolese susceptible to waterborne diseases in addition to COVID-19.^9^ Another problem is a shortage of healthcare workers. According to the WHO,^10^ there are only 0.28 physician per 10,000 population, which is far below the required goal of 22.8 professional health workers per 10,000. Embezzlement of pandemic response funds left the health workers unpaid for months, which forced them to run a partial strike in Kinshasa.^11^ Corruption can discourage health workers and contribute to low-quality health services.

The DRC is one of the poorest countries in the world, where more than 70% of the people live in poverty.^12^ Lack of government assistance for those who had lost their jobs during the pandemic-related lockdown forced people to look for provisions outside their houses, thus contributing to spread of the virus. In addition to insufficient testing capacity, lack of belief in the reality of the virus,^3^ stigma, and fear of quarantine caused people not to undergo testing, resulting in underreporting the true number of cases. Untrustworthy sources deliver misinformation and rumor to people and make them vulnerable to the disease.^13^ Other concerning issues that will contribute to surge of the cases are porous borders and large gatherings of people in marketplaces and places of worship as the restrictions have been lifted.^9^

The DRC is home to many rebel groups violating human rights by killing and displacing people and looting their houses as well as health facilities for decades. They also target health workers and discourage their activities. Restrictions had created a glimmer of hope to pause the conflicts and allow Congolese to receive the needed healthcare services. However, the country continues to experience the conflicts in the country, primarily in eastern regions, which have resulted in hundreds of thousands of internal displacements, hampering social distancing and causing an additional burden for the healthcare system. This year, in Ituri Province, in the north east of the country, at least 1,315 have been killed, including 165 children and 300,000 people were displaced as of August 14, 2020.^14^ Before the pandemic, this region hosted 1.2 million internally displaced people in 2019. Moreover, thousands have been forced to seek refuge in neighboring countries.^14^ The Save the Children warns that displaced people do not have access to sufficient food. These violence have also cut off the area and led to the cessation of health and nutrition services as well as clean water and hygiene materials in the province, resulting in 235 new cases of severe acute malnutrition in July 2020 that are left without follow-up and treatment because of the continuing violence, which may lead to deaths.^14^ According to UNICEF, 10 health facilities have been damaged and 18 others abandoned by health workers escaping the violence in the South-Kivu Province,^15^ depriving many people of basic health services.

Throughout the DRC, especially in the east, many vulnerable people to COVID-19 live who suffer from preconditions such as diabetes, high blood pressure, and also fatal diseases and epidemics such as measles, malaria, cholera, acute respiratory infections, HIV/AIDS, tuberculosis, malnutrition, and Ebola.^13^ These diseases and epidemics, especially Ebola intertwine to cripple the fragile healthcare system of the country. The DRC is the epicenter of Ebola which has a high fatality rate.^16^ Ebola epidemics have occurred periodically and have taken the lives of thousands of Congolese. While the country was on the verge of announcing an end to its 10th outbreak, the 11th epidemic emerged in Equateur Province in the west of the DRC on June 1, 2020. Since then, it has been increasing at alarming pace, with 102 confirmed cases and 44 deaths in less than 3 months.^16^ Three of those confirmed cases are among health workers as of September 1, 2020. Ebola cases are expected to grow, with shortfalls of funds and insufficient numbers of doctors exacerbating the situation. According to the WHO, the cumulative funding requirement for control of Ebola from January to September 2020 is 28.5 million United States dollars; however, they have received only 6,849,693 USD.^17^

## CONCLUSION

The DRC healthcare system has many formidable challenges such as COVID-19, protracted conflicts, and deadly epidemics and diseases. These challenges deter containment efforts and pose further burdens on an already weak healthcare system. Thus, the government, with the cooperation of regional powers, needs to address them similarly to the way they overcame the previous Ebola epidemics. The government should have a balance in shifting the available resources between COVID-19 and other diseases. Until a vaccine is available, the DRC needs to be prudent when lifting restrictions to prevent explosion of new cases in its second wave.
